# Utilizing Laser-Induced Fluorescence to Evaluate the Efficiency of Carbon Dioxide and Diode Lasers in Dentin Sealing after Tooth Preparation: An in-vitro Study

**DOI:** 10.1007/s10895-024-03816-4

**Published:** 2024-07-10

**Authors:** Asmaa K. Abo-ELsouood, Haythem S. Moharrum, Ahmed Abbas Zaky, Omnia Hamdy

**Affiliations:** 1 Department of Medical Applications of Laser, National Institute of Laser Enhanced Sciences (NILES), Cairo University, Giza, 12613 Egypt; 2 Department of Engineering Applications of Laser, National Institute of Laser Enhanced Sciences (NILES), Cairo University, Giza, 12613 Egypt

**Keywords:** LIF, CO2 laser, Immediate dentin sealing, Adhesive, Impression material

## Abstract

Adhesive dentistry has made it feasible to design restorations with high aesthetic qualities and little invasiveness. However, the freshly cut dentin after the tooth preparation needs to be sealed to prevent its contaminations, bacterial leakage, and hypersensitivity. Consequently, the immediate dentin sealing (IDS) method has been advised. This study examines different dentinal tubule sealing methods via CO2 laser, diode laser (980-nm) and a two-step self-etch adhesive system applied directly to the fresh cut dentin preceding the placement of the provisional phase. The sealing efficiency of each laser and bond system was evaluated based on the laser-induced fluorescence (LIF) properties and image analysis by scanning electron microscopy. Moreover, the obtained LIF spectra were evaluated using partial least square progression. A two-step adhesive containing a high concentration of S-PRG fillers produced a thick layer that was not perfectly uniform at all sites due to uneven filler distribution in the bond with totally and partially closed dentinal tubules. However, the peaks of the LIF spectra dropped after applying laser because of its sealing effectiveness. Accordingly, CO2 and diode lasers have strong evidence in dentinal tubule sealing and a definitive treatment modality for dentinal hypersensitivity. Moreover, IDS with an adhesive system is superior in occluding dentinal tubules in a biomimetic manner based on its filler content and bioactive properties.

## Introduction

Destruction of tooth tissue is not supported by restorative dentistry. As a result, restorations with a low degree of invasiveness are employed, including laminate veneers, inlays, and onlays. No matter how much of the tooth’s material is removed, exposure still happens [1–3]. There will always be dental tubules. It is typical for the patient to have uncomfortable symptoms throughout the provisional phase and after the final restoration is completed. This unpleasant symptom is characterized by a brief, severe pain in reaction to heat and chemical stimulation. Numerous factors, including preparation-related warming and dehydration, bacterial micro-leakage, and fluid migration via dentin tubules, might be blamed for this outcome [4].

In order to overcome the challenges and prevent potential pulp injury, Ashley et al. [5] invented the dentin-bonding agent (DBA) in the early 1990s, right after tooth preparation and before taking an impression. Dentinal tubules are sealed using conventional methods (delayed dentin sealing [DDS]) during the final restoration’s bonding step. Therefore, exposed dentin during provisionalization presents a potential entry point for bacterial invasion. The IDS (instant dentin sealing) procedure, in contrast, applies dentin adhesives before the provisional phase, which has benefits in terms of bacterial microleakage, dentin hypersensitivity [6], gap development, and bond strength [7]. According to researchers, two-step selfetch systems and older three-step etch-and-rinse systems perform better than single-step systems in terms of durability, ageing, and bond strength [8, 9].

Implementing IDS rather than DDS seems to provide mainly advantages. However, there have been complaints of difficulties with impressions on teeth that have undergone the IDS procedure. The impression material could react with the top resin layer. This outer layer, called the oxygen-inhibition layer (OIL), did not seem to polymerize, which might have an impact on the imprinting process [10]. The cleaning procedure has the most impact on the leftover impression material [11]. Utilizing laser technology is another therapy approach for lowering dentinal hypersensitivity. The initial laser procedure was completed. Nd: YAG laser therapy was employed by Matsumoto et al. (1985) to treat dentin hypersensitivity [12, 13]. The desensitizing method varies based on the type of laser being used, the irradiation parameters (sufficient wavelength, power density, wave-mode, frequency of pulses, and number of irradiation repetitions), and the success rates that were observed. Numerous studies have characterized the impacts of lasers in various ways. Scanning electron microscopy (SEM) was used to examine the in vitro mechanism of high-level lasers in desensitization after irradiation [14, 15].

Numerous studies have discovered dentinal tubule occlusion, suggesting that this may be a mechanism for pain relief. Laser irradiation may enhance the effects of normal desensitization therapy in occluding tubules [15, 16]. Several researches have looked at how 810-nm, 980-nm, and 10,600-nm-CO2 lasers can shrink the diameter of dentinal tubules [17–19]. Pathogenicity in dental hard tissue is more accurately assessed by laser induced fluorescence analysis [20, 21]. Laser-induced fluorescence (LIF) spectroscopy emerges as a promising optical technique with extensive applicability in medical and industrial domains [22, 23]. Its versatility has been demonstrated in various fields, including biological research, tumor classification [24], detection of food adulteration [25], and quality inspection [26]. In order to enhance the accuracy of early diagnoses and secondary caries prevention, investigations are currently focused on learning more about the disorganization of a native dentin matrix [27]. Depending on the makeup of the dental tissue, the fluorescence ratios demonstrated large variations [28].

The present study compares several adhesive sealing (IDS) technologies via laser based on the results obtained from typical laser-induced fluorescence (LIF) analysis. We used a thorough data analysis plan in our work to assess the properties of LIF and image analysis from scanning electron microscopy (SEM). The main goal was to use CO2 and diode lasers to assess the sealing efficacy of various IDS methods. The observed LIF spectra of each sample group were evaluated by statistical analysis using partial least squares regression (PLSR). Furthermore, we used scanning electron microscopy (SEM) with an attached EDX Unit (Energy Dispersive X-ray Analyses) to examine the samples’ morphological structures under specified operating settings.

## Materials and Methods

### Samples Collection and Preparation

For orthodontic reasons, sixty premolar teeth were extracted and divided randomly into six groups. To participate in the trial, all patients had to be clinically healthy and free of any acute general disease. To achieve uniform grinding speed and torque, the buccal and palatal dentine of upper premolars or the buccal and lingual dentine of lower premolars were exposed using a diamond bur (medium grit) on a hand drill with water spray at 30,000 revolutions/min (Dentsply Sirona, Germany) to replicate the best standard clinical methods [23]. After preparation, the teeth surfaces were finished with red coded finishing bur and polished with rubber cups under usage of coolant then cleaned with a water spray for 30 s, and lightly dried with air at room temperature. The teeth are divided into six groups:

#### Group I (Control)

Polyvinyl siloxane impression material (PVS; Aquasil Ultra-fast set XLV; Dentsply Sirona, York, PA, USA) was injected into the prepared surfaces of the specimens and then softly spread over the impression surfaces using gentle air pressure. Following the manufacturer’s instructions, the material was allowed to solidify normally, as in the clinical context, as demonstrated in [29].

#### Group II (Diode Laser)

Specimens treated with a 1 W, 980 nm diode laser for 20 s in continuous, tangential, noncontact mode (the optical fiber was 1 mm away from the irradiated surface). The optical fiber had a diameter of 320 μm. The irradiation rate was 1 mm/sec. Specimens were put on a flat surface, and the optical fiber was moved tangentially (at a 45-degree angle) at *1 mm/sec speed by the operator [17]. Then polyvinyl siloxane impression material (PVS; Aquasil Ultra-fast set XLV; Dentsply Sirona, York, PA, USA) was injected into the prepared surfaces of the specimens and then softly spread over the impression surfaces using gentle air pressure. Following the manufacturer’s instructions, the material was allowed to solidify normally, as in the clinical context.

#### Group III (CO2 laser)

Specimens treated with a 1 W, 10,600 nm CO2 laser with continuous pulse mode; exposure time:20s; distance:1 cm; non-contact mode with wet conditions [30]. Then specimen injected with polyvinyl siloxane impression material (PVS; Aquasil Ultra-fast set XLV; Dentsply Sirona, York, PA, USA) was injected into the prepared surfaces of the specimens and then softly spread over the impression surfaces using gentle air pressure. Following the manufacturer’s instructions, the material was allowed to solidify normally, as in the clinical context, as demonstrated.

#### Group IV (Bonded)

Specimens were cleaned with phosphoric acid at 37% for 5 s, then with water spray for 20 s, slightly dried with air, and treated with FL-BOND II (Shufo, Japan), After actively applying self-etch primer to dentin for 20 s (“dentin spa”), solvents are evaporated with gentle air-drying for 5 s. The lightly packed adhesive is air-thinned for 5 s and light-polymerized for 15 s [9] using glycerin jelly to block air [10] following the manufacturer instructions [2]. Then injected with polyvinyl siloxane impression material (PVS; Aquasil Ultra-fast set XLV; Dentsply Sirona, York, PA, USA) was injected into the prepared surfaces of the specimens and then softly spread over the impression surfaces using gentle air pressure.

#### Group V (Bonded + CO2 Laser)

Specimens were cleaned with phosphoric acid at 37% for 5 s, cleaned with water spray for 20 s, slightly dried with air, and treated with FL-BOND II (Shufo, Japan), After actively applying self-etch primer to dentin for 20 s, solvents are evaporated with gentle air-drying for 5 s, The lightly packed adhesive is air-thinned for 5 s and light-polymerized for 15 s, using glycerin jelly following the manufacturer instructions then specimens treated with a 1 W,10,600 nm CO2 laser with continuous pulse mode; exposure time:20s; distance:1 cm; non-contact mode with wet conditions. Then injected with polyvinyl siloxane impression material (PVS; Aquasil Ultra-fast set XLV; Dentsply Sirona, York, PA, USA) was injected into the prepared surfaces of the specimens and then softly spread over the impression surfaces using gentle air pressure.

#### Group VI (Bonded + Diode Laser

Specimens were cleaned with phosphoric acid at 37% for 5 s, cleaned with water spray for 20 s, slightly dried with air, and treated with FL-BOND II (Shufo, Japan), After actively applying self-etch primer to dentin for 20 s, solvents are evaporated with gentle air-drying for 5 s, The lightly packed adhesive is air-thinned for 5 s and light-polymerized for 15 s, using glycerin jelly following the manufacturer instructions then specimens treated a 1 W,980 nm diode laser for 20 s in continuous, tangential, noncontact mode (the optical fiber was 1 mm away from the irradiated surface). The optical fiber had a diameter of 320 μm. The irradiation rate was 1 mm/sec. Specimens were put on a flat surface, and the optical fiber was moved tangentially (at a 45-degree angle) at *1 mm/sec speed by the operator. Then injected with polyvinyl siloxane impression material (PVS; Aquasil Ultra-fast set XLV; Dentsply Sirona, York, PA, USA) was injected into the prepared surfaces of the specimens and then softly spread over the impression surfaces using gentle air pressure.

### LIF Optical Setup

**Fig. 1:**
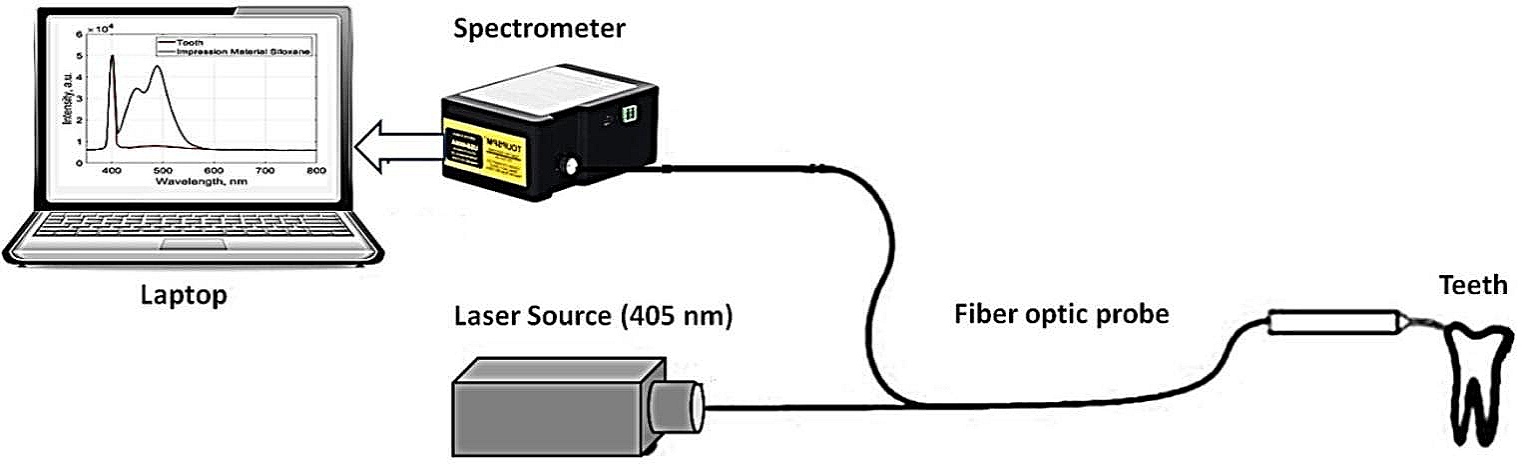
LIF experimental configuration

The samples were quantitatively evaluated using LIF spectroscopy. The excitation laser source is a continuous-wave diode pumped solid-state (DPSS) laser source (XL-R405SD, Xinland international Co., Ltd, China) at wavelength of 405 nm and output power of 100-mW. Around 2 mm is the size of a laser beam. The measuring detector (Toshiba TCD1304AP Linear CCD array (Sony ILX511 2048 Linear CCD array)) has a response range of 200–1100 nm wavelengths, 3648 pixels, and 8 × 200 μm pixel size. Data processing and analysis were carried out using the spectrometer software (Toup Spm) and Matlab R2018b. Fluorescence spectra were captured using point monitoring when the hand piece was in contact with the lesion surface. Schematics of the LIF system optical setup are shown in Fig. 1.

### Partial Least Squares Regression (PLSR)

To statically assess the measured LIF spectra of each sample group, partial least squares regression (PLSR) (a multivariate calibration technique) was employed. Applying PLSR, new predictors known as “components” are created to model the response variables of datasets with a large number of predictor variables that are highly correlated [31]. The response variable is taken into consideration while building the PLSR components illustrating the observed predictors’ variability [32, 33]. PLSR is a powerful tool for modeling the relation between predictor factors (LIF spectra) and response variables (sealing efficiency). Using this technique, we were able to extract useful information from the complicated LIF spectra and assess the efficiency of each laser and bond system in closing dentinal tubules. We were able to assess the sealing efficacy by comparing the changes in the LIF spectra peaks before and after laser application. The standard MATLAB function “plsregress” was used to implement PLSR of the recorded LIF spectra in the current investigation [34]. Determining the PLS component count is the first stage in the implantation process. After computing the predictor and response loadings, partial least-squares regression of the response matrix on the predictor variable matrices is performed. The R-squared (R^2^) value is also estimated.

### Scanning Electron Microscopy (SEM)

Samples were fixed on aluminum stubs with standard diameter using a carbon double sticky tape. Then the samples were coated with gold using sputter coater modal EMITECH, K550X England. Scanning electron microscope (Model Quanta 250 FEG – made in Holland) attached with EDX Unit (Energy Dispersive X-ray Analyses) was employed to investigate the morphological structures of the samples under operating conditions of accelerating voltage 20 K. V, resolution for Gun. 1 nm, and magnification x500, x2000 and x5000. Representative images of different samples were selected. The SEM study was conducted to determine the penetration of the impression material into the dentinal tubules. The presence of material tags and remains was detected and examined using image analysis. On the other hand, histogram analysis was performed on SEM images of the treated dentin surfaces with the adhesive bond to evaluate the remaining unsealed dentinal tubules with bond fillers. Using these separate approaches (i.e. image analysis and histogram analysis) for the non-bonded and bonded groups, we were able to analyze impression material penetration in non-bonded samples and adhesive system sealing efficacy in bonded samples. This allowed us to compare and evaluate the efficacy of various dentinal tubule sealing techniques.

## Results and Discussion

### LIF Spectra

**Fig. 2:**
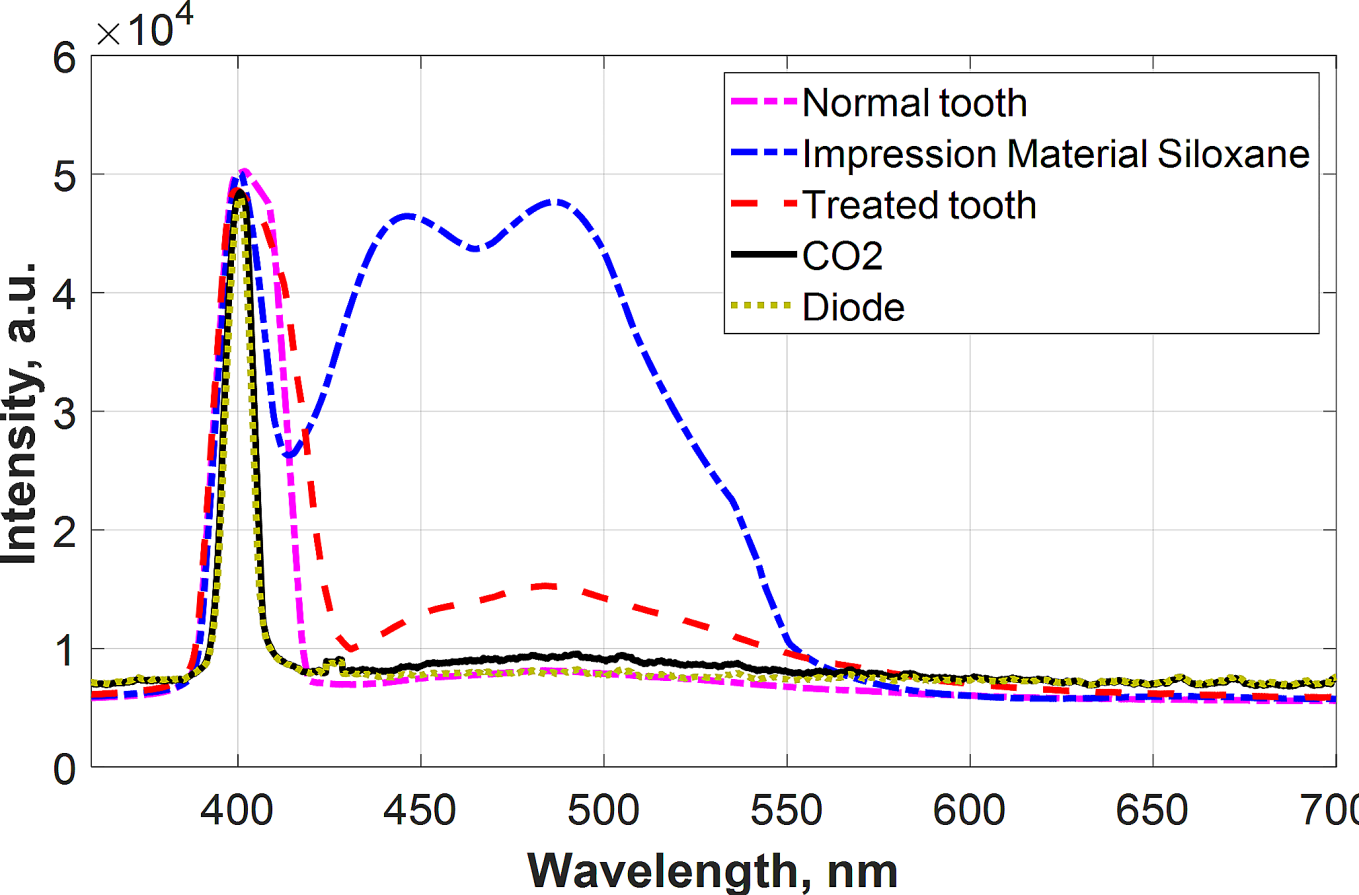
The recorded LIF spectra for normal tooth, impression material siloxane only and treated tooth followed by laser application

The current study identified various materials based on their fluorescence emission properties. The obtained LIF spectra from investigated surfaces are presented in Fig. 2. The highest fluorescence peak was recorded for impression material. It is evident that applications of different laser types (10,600 nm CO2, 980 nm diode laser) show decreasing in the fluorescence intensity.

**Fig. 3:**
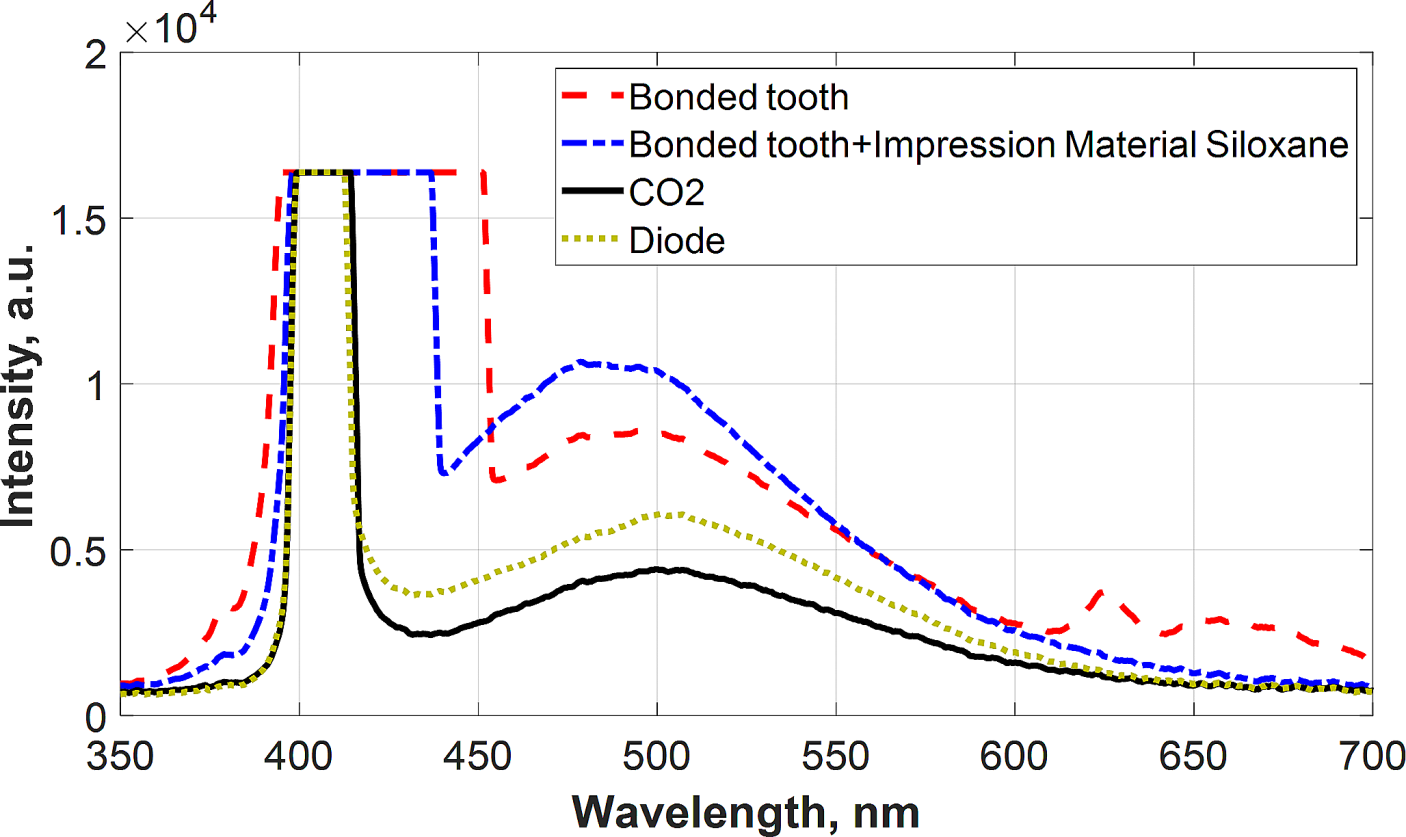
The recorded LIF spectra for bonded tooth followed by laser application

As shown in Fig. 3, the highest emitted fluorescence peak was obtained from the tooth treated with the bond and then injected with polyvinyl siloxane impression material. The CO2 and diode treated tooth surfaces showed the least fluorescence peaks however the diode laser fluorescence peak was a little shorter than CO2.

### PLSR Analysis

**Fig. 4:**
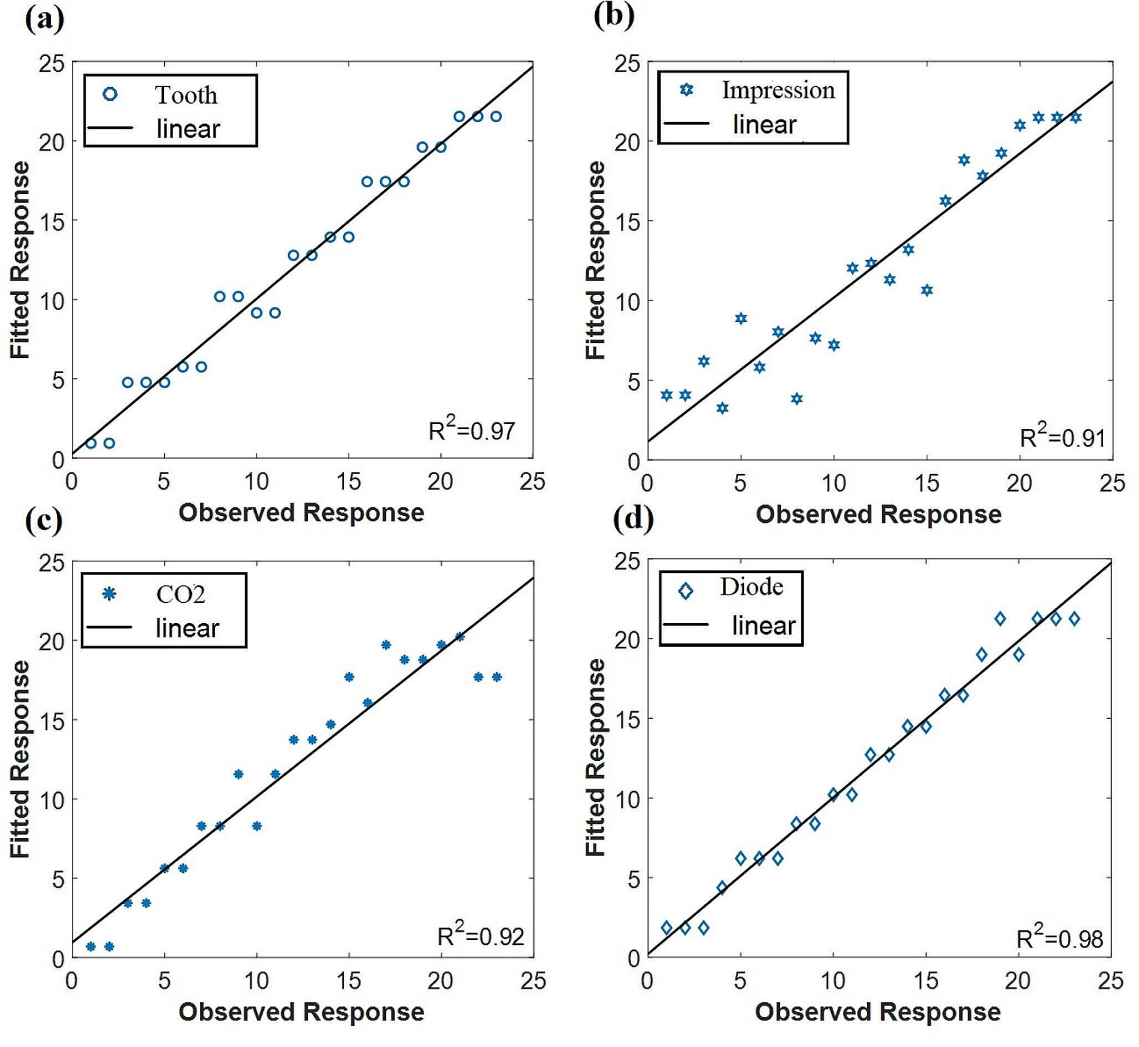
PLSR results of the obtained LIF spectra of tooth before and after using impression material siloxane

The PLSR approach was used to statistically evaluate the fluorescence data before and after employing the investigated materials (i.e., impression or bond) and the laser treatment. Through PLSR correlation was assessed. The relation between the experimental values and predicted values according to measured spectroscopic data was measured is shown in Fig. 4 the calculated R^2^ of 0.97, 0.91, 0.92, 0.98 shows very good model performance. The best performance model was obtained from a diode laser which is nearly approximate to natural tooth response.

**Fig. 5:**
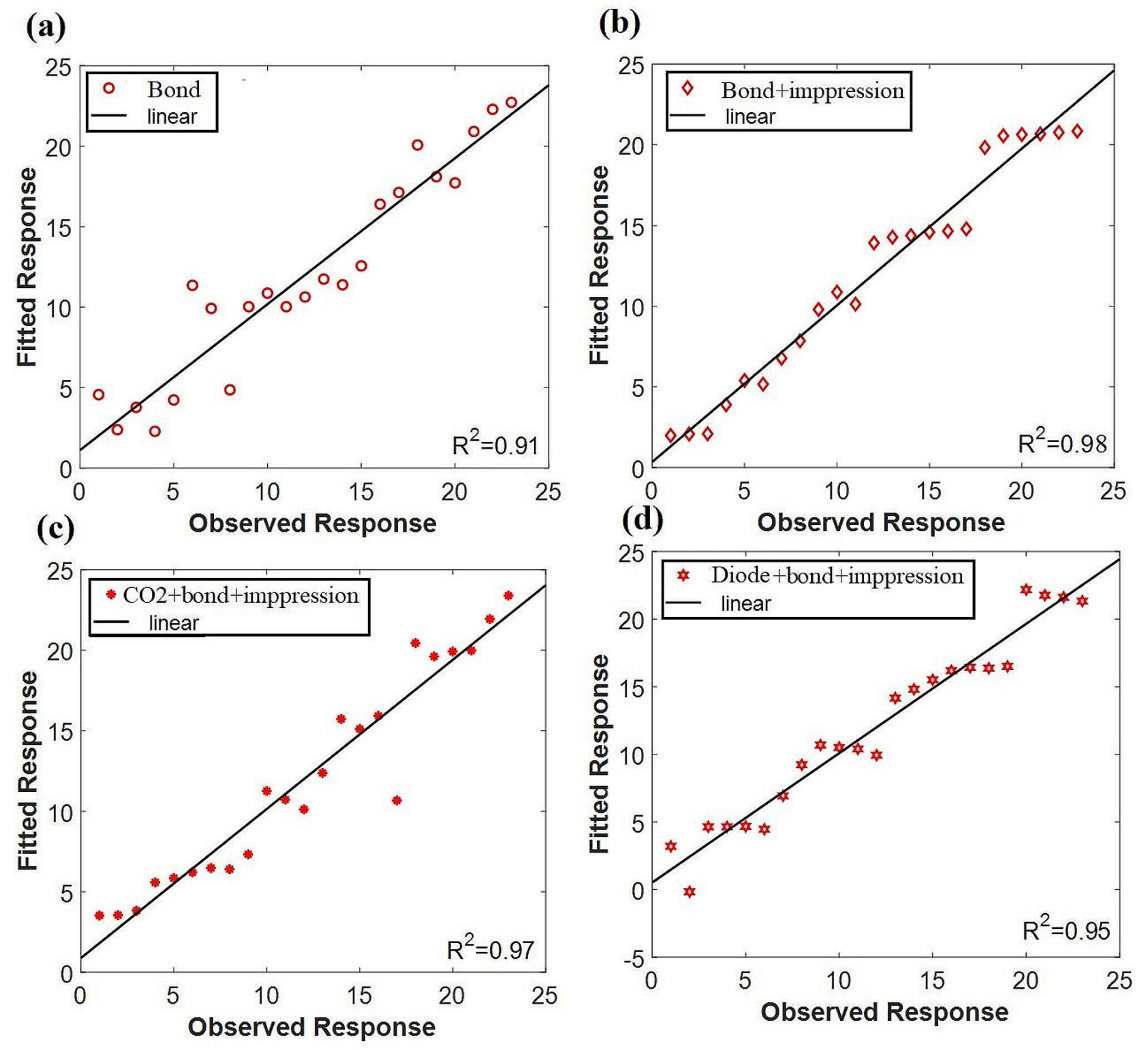
PLSR results of the obtained LIF spectra of tooth before and after using both bond and impression material siloxane

In Fig 5 the observed response R^2^ of 0.98 in the (bonded + impression) group showed a very good performance model and is close to R^2^ of CO2 and diode groups which are 0.97, 0,95 respectively.

### SEM Image Analysis

Depending on whether adhesive was used or not, we separated the SEM study into two distinct analysis methodologies with constant magnification (5000X), as the bond layer affects the features of the images in different ways.

#### Percentage of Material Tags Area

**Fig. 6:**
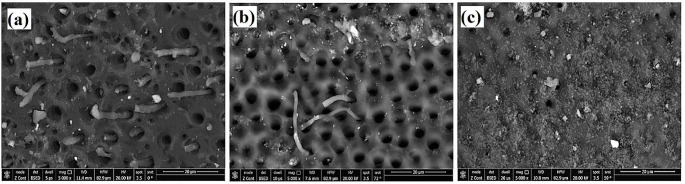
SEM images for (**a**) group I (control), (**b**) group II (diode Laser), (**c**) group III (CO2 laser)

The first 3 groups were analyzed by SEM to determine the penetration of the impression material inside the dentinal tubules. No remnants of impression material appeared with the naked eye. Under the magnification of SEM, the material tags and remnants were observed in the first three groups (not bonded) shown in Fig. 6. Image analysis was performed by dividing the percentage of material tags area by the total area of the image at a magnification of 5000X as reported in Table 1.

**Table 1:** Image analysis according to impression tags area using total image area of 4497.881 (µm^2^)

	Image	Resin tags area (µm^2^)	%	Mean	SD
**Normal**	001_001	369.176	8.21	7.90	0.94
	001_011	388.17	8.63		
	001_005	307.838	6.84		
**Diode**	001_021	162.43	3.61	4.48	1.22*
	001_016	178.173	3.95		
	001_023	264.571	5.88		
**CO2**	001_037	77.075	1.71	2.21	0.54**
	001_038	125.45	2.79		
	001_039	95.88	2.13		

Unpaired *t* test compares the means of two groups significantly different at *P value < 0.01 and **P value < 0.001

#### Image Histogram

The other three groups (bonded) showed representative SEM images of the treated dentin surfaces with bond after tooth preparation for groups IV, V, and VI. The SEM images show high sealing and blockage of mostly dentinal tubules with a thick coat of the adhesive containing S-PRG fillers. Because of the thick bond layer, the histograms of the images analyze the remaining unsealed dentinal tubules with the bond fillers. A histogram is a graphical representation of data that shows how light intensity data is manifested in pictures. When a picture is represented in grayscale, a histogram shows the frequency of each intensity value from 0 to 255. Every intensity value and the quantity of pixels with that value are plotted. It is crucial theoretically because it helps us understand how different intensities are distributed throughout an image. The histogram’s color scale begins with black on the left and gradually changes to white as you approach towards the right. As a result, a histogram shows blacks, shadows, mid tones, highlights, and whites as the graph advances from left to right.

## Discussion

**Fig. 7:**
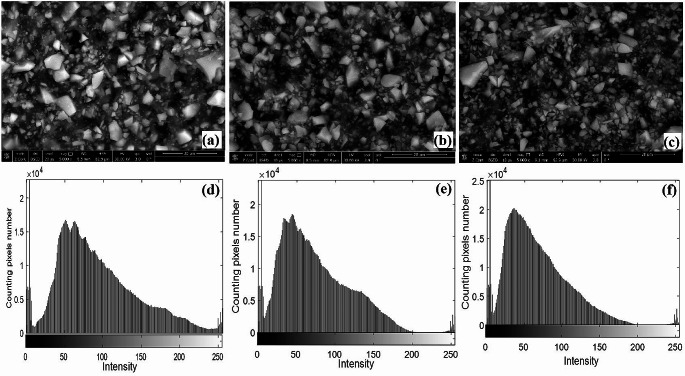
SEM images for *g* roup IV, V, and VI under SEM 5000x

Numerous studies have demonstrated that it is crucial that the adhesive substrate not be contaminated to have good adhesion between resin and tooth and, subsequently, for a healthy restoration. Blood, gingival fluid, saliva, and lubricating oils are among the substances that can shorten the life of restorations and cause pigmentation, caries, detachment, and sensitivity of the substrate [35]. Because of the possibility of elastic deformation during removal, it is now understood that the interface area between the impression material and the tooth is the most challenging to control [36]. This is why one of the most researched rheological properties of impression materials is the characteristics of elastic deformation during the phase of impression removal [37]. In this study, different approaches propose sealing the tubules immediately after preparation. Lasers have already proven to be proficient at treating dentin hypersensitivity [38]. This study is the first to have used LIF spectra for polyvinyl siloxane impression material which indicated high fluorescence properties presented by the highest LIF peak. Lowering of LIF peaks when applying 980 nm diode laser and 10,600 nm CO_2_ laser on the tooth surface due to their sealing efficacy [39, 40] hinders the contamination of the impression entrapped in tooth surface and dentinal tubules lumen. In 2020, Sinjari et al. [41] demonstrated the penetration of impression material into a freshly cut dentin which supports this study result. Under SEM the impression tags appear in all experimental non-bonded samples which promote the benefits of IDS with the adhesive system to dentin directly after tooth preparation, before impression which can be filled or unfilled with a resin coat [9]. A review study combining numerous articles reported on and addressed the effects of S-PRG fillers on enamel, dentin, and root cavities which mainly used in dental varnish and adhesives [42]. Application of a two-step adhesive with a high concentration of S-PRG fillers resulted in a thick coat that wasn’t precisely uniform at all points due to uneven filler distribution in the bond with completely and partially plugged dentinal tubules, as determined by histogram analysis in Fig. 7.

## Conclusions

Under in vitro conditions, laser-induced fluorescence with laser sources at (405 nm) and SEM has been utilized within the framework of the integrated measuring system to reveal the immediate sealing efficacy of different methods. Comparing the results obtained in our work with those known from scientific information demonstrated that although CO2 (10,600 nm) and diode (980 nm) lasers have strong evidence in dentinal tubule sealing and a definitive treatment modality for dentinal hypersensitivity after tooth preparation, IDS with an adhesive system is superior in occluding dentinal tubules according to its filler content and bioactive properties in a biomimetic manner. The selection of filled resin adhesive that can produce a thick adhesive layer appears to contribute to the success of the IDS technique.

## Data Availability

No datasets were generated or analysed during the current study.
